# Screening for *C. elegans* male copulation-defective mutants by the mating plug phenotype

**DOI:** 10.17912/W2SS9K

**Published:** 2017-11-02

**Authors:** Katharine Liu, Yvonne Hajdu-Cronin, Ann Chen, Gary Schindelman, Allyson Whittaker, Shahla Gharib, Paul Sternberg

**Affiliations:** 1 California Institute of Technology, ​Division of Biology and Biological Engineering, Caltech, Pasadena CA 91125

## Description

We describe an efficient screen for male mating defective mutants in *Caenorhabditis elegans*. We report the isolation of 20 alleles that confer specific mating defects. In a previously reported screen (Hajdu-Cronin et al, 2017), we isolated 19 Cod (for copulation defective) strains in which morphologically wild-type males fail to mate. Failure to mate could conceivably result from defects in any step of the mating process: response, turning, vulva location, spicule insertion, and sperm transfer. By observation, we identified mutants defective in each of these steps except for vulva location. We believe that this was due to both redundancy of sensory structures mediating this step and our stringent screening conditions. To address this, we modified the Cod screen, using the strain *plg-1*(*e2001d*); *him-5*(*e1490*), in which the presence of a copulatory “plug” over the hermaphrodite vulva provides a visible marker for successful mating (Hodgkin and Doniach, 1997). We backcrossed *plg-1*(*e2001d*) four times into *him-5*(*e1490*) to make strain PS1395, the parent of our initial screen (*sy4xx* series). We subsequently repeated our screen after re-isolating a *plg-1*(*e2001d*); *him-5*(*e1490*) strain PS3696 that had consistent mating behavior and brood size (PS3696 is the parental background for the s*y6xx* series). Since we select for whether males are able to mate with their moving siblings as opposed to paralyzed hermaphrodites, we expected to isolate more subtle Cod mutants (such as incompletely penetrant vulva location defects). The screen would also allow for the identification of plug formation defective and hermaphrodite-specific mating defective strains. In one PS3696 screen, of 1400 F2 clones, 5% were non-*Plg*, 280 were then examined for behavior; we kept 69 as candidates; eight had strong phenotypes and normal morphology and were given allele names (*sy678, sy680, sy681, sy682, sy683, sy684, sy685,* and *sy678*). *sy681* turned out to have the same molecular lesion as *sy680* and was discarded. Overall, we isolated 20 Cod mutants from several screens including several pilot screens (Table 1). *sy671* was isolated in this screen and found to be an allele of *unc-18*(Schindelman et al., 2006).

**Table 1**

**Table:** 

**Allele**	**Isolation name**	**Phenotype**	**Gene**
sy414		Turning; also Egl	
sy416	2.2.6	Response	
sy419	18.6.6	Vulval location, low penetrance	*cod-12*
sy420	9.15.5	Vulval location	*cod-13*
sy421	4.7.1	Vulval location	*cod-14*
sy422	13.14.1	Vulval location	*cod-14*
sy423	21.14.1	Vulval location	*cod-15*
sy430	6.20.2	Spicule insertion	
sy431	13.13.8	Spicule insertion	
sy671	336.5	Sperm transfer initiation	*unc-18*
sy672	801.6	Sperm transfer continuation	
sy678	247.1	Multiple mating steps	
sy680	655.1	Response, Vulval location	*pkd-2*
sy681	627.3	Response, Vulval location	*pkd-2*
sy682	1358.2	Response, Vulval location	*lov-3*
sy683	1345	Response, Vulval location	
sy684	179.6	Response, turning, vulval location	
sy685	740.5	Response and turning	
sy709	1263.8	Male-specific coiler	

## Reagents

**ReagentsStrains:PS1861:** *cod-12(sy419);* *plg-1(e2001d);* *him-5(e1490)***PS1862:** *sy416;* *plg-1(e2001d);* *him-5(e1490)***PS2011:** *cod-14(sy421);* *plg-1(e2001d);* *him-5(e1490)***PS2012:** *cod-14(sy422);* *plg-1(e2001d);* *him-5(e1490)***PS2013:** *cod-15(sy423);* *plg-1(e2001d);* *him-5(e1490)***PS2118:** *sy430;* *plg-1(e2001d);* *him-5(e1490)***PS2128:** *sy431;* *plg-1(e2001d);* *him-5(e1490)***PS3696:** *plg-1(e2001d);* *him-5(e1490)***PS4219:** *plg-1(e2001d);* *him-5(e1490); sy672***PS4769:** *plg-1(e2001);* *him-5(e1490)sy709***PS5421:** *plg-1(e2001d); him-5(e1490)sy678***PS5422:** *plg-1(e2001);* *him-5(e1490); sy684***PS6218:** *sy685; plg-1(e2001);* *him-5(e1490)***PS1972:** *sy414; plg-1(e2001d);* *him-5(e1490)***PS4770:** *plg-1(e2001d);* *him-5(e1490); sy682***PS1860**: *cod-13(sy420);* *plg-1(e2001d);* *him-5(e1490)***PS1395:** *plg-1(e2001d);* *him-5(e1490)*
